# Beyond Tumor Suppression: The Multifaceted Functions of HOPX in Tissue Differentiation, Metabolism, and Immunity

**DOI:** 10.3390/cells14211718

**Published:** 2025-11-01

**Authors:** Fabian Munzert, Miljana Nenkov, Alexander Berndt, Tim Sandhaus, Susanne Lang, Nikolaus Gaßler, Yuan Chen

**Affiliations:** 1 Section Pathology of the Institute of Forensic Medicine, Jena University Hospital, Friedrich Schiller University Jena, Am Klinikum 1, 07747 Jena, Germany; fabian.munzert@uni-jena.de (F.M.); miljana.nenkov@med.uni-jena.de (M.N.); alexander.berndt@med.uni-jena.de (A.B.); nikolaus.gassler@med.uni-jena.de (N.G.); 2 ThIMEDOP—CeTraMed, Jena University Hospital, Friedrich Schiller University Jena, Am Klinikum 1, 07747 Jena, Germany; 3 Clinic of Cardiothoracic Surgery, Jena University Hospital, Friedrich Schiller University Jena, Am Klinikum 1, 07747 Jena, Germany; tim.sandhaus@med.uni-jena.de; 4 Department of Internal Medicine V, Jena University Hospital, Friedrich Schiller University Jena, Am Klinikum 1, 07747 Jena, Germany; susanne.lang@med.uni-jena.de

**Keywords:** HOPX, homeodomain protein, tissue differentiation, metabolism, tumor suppression, immunity, tumor immunotherapy

## Abstract

The transcription factor homeodomain-only protein X (HOPX) is the smallest member of the homeodomain protein family. Lacking a DNA-binding domain, it acts as a co-effector, interacting with other transcription factors such as serum response factor (SRF) and GATA-binding factor 6 (GATA6) to regulate the differentiation and development of the heart and lung. HOPX exerts a tumor-suppressive function in various types of epithelial-derived carcinoma, while it promotes oncogenic effects in mesenchymal-derived sarcoma, indicating a distinct role of HOPX in the two major types of the malignancy. In addition, accumulating evidence shows that HOPX is expressed in the immune system and involved in the differentiation of immune cells. Recently, the emerging role of HOPX in metabolism has gained attention. This review describes the identification of HOPX in various tissues and discusses its role in carcinogenesis, as well as its functions in tissue differentiation, lipid metabolism, immunity, and the tumor microenvironment. The participation of HOPX in carcinogenesis and immunity implies that it may serve as a potential enhancer in tumor immunotherapy.

## 1. Introduction

Homeodomain-containing genes consist of a specific 183-base-pair DNA sequence encoding a homeodomain of 61 amino acids, which functions to recognize and bind to sequence-specific DNA motifs [1]. Homeodomain genes have been identified across a wide range of species, including Drosophila, insects, reptiles, cnidarians, frogs, mice, and humans, as well as non-animal species like fungi and plants [2]. To date, several common types of homeodomain genes, such as *homeobox* (*HOX*), *empty spiracles homeobox* 1 (*EMX*), *paired box* (*PAX*), and *Msh Homeobox 1* (*MSX*), together with many divergent homeodomain genes, have been characterized [3]. Different types of homeodomain genes exert distinct biological functions. The common feature is that the majority of the homeodomain genes are transcription factors, playing crucial roles in organogenesis and pattern formation during embryogenesis, cell differentiation, vascular remodeling, and angiogenesis [4]. Abnormalities in the homeodomain proteins are closely associated with various human diseases, including developmental disorders, congenital diseases, and tumors.

In addition, evidence supports the involvement of the homeodomain proteins in regulating metabolism. For instance, the specific homeodomain proteins islet/duodenum homeobox-1 (IDX-1) and presequence protease 1 (PREP1) directly influence glucose and lipid metabolism and participate in the pathogenesis of insulin resistance and diabetes [5,6]. The TG interacting factor 1(TGIF1) protein, a member of the TALE (three-amino-acid loop extension) superfamily of homeodomain proteins, represses cholesterol acyltransferase 2 (SOAT2), an enzyme involved in the synthesis of cholesterol esters from cholesterol and fatty acids, to modulate lipid metabolism [7]. Also, HOX proteins are actively expressed in human adipose tissues and regulate lipid metabolic processes [8].

The immune microenvironment influences the progression and manifestation of various diseases through its complex regulatory network [9]. In the context of tumors, immunological heterogeneity is present spatially and temporally, associated with tumor progression and therapy responses, in particular immunotherapy responses [10]. Immunotherapy targeting the programmed cell death protein 1/programmed cell death ligand 1 (PD1/PD-L1) and cytotoxic T-lymphocyte-associated protein 4 (CTLA-4) immune checkpoint signaling has proven to be successful in the treatment of various metastatic tumors [11]. Tumor mutational burden (TMB), PD-L1 expression, the gut microbiome, and molecular and cellular features within the tumor microenvironment (TME) are related to therapeutic outcomes [12]. In addition, recent advances in tumor immunotherapy strategies targeting natural killer (NK) immune checkpoints have shown considerable promise [13]. Recently, the role of the homeodomain proteins in tumor immunity and immunotherapy has attracted attention. In pediatric gliomas, the homeobox-related gene signature, including *HOXA6*, *HOXC4*, *HOXC5*, *HOXC6*, and *HOXA-AS3*, predicts prognosis and immune infiltrate characteristics in the immune microenvironment [14]. Double homeobox protein 4 (DUX4) is characterized as a common driver of immune exclusion and contributes to a decreased objective response to anti-PD-L1 therapy in urothelial carcinoma patients [15]. In addition, hematopoietically expressed homeobox protein (Hhex) represses Bcl-2 interacting mediator (BIM)-dependent apoptosis to enhance NK cell survival and maturation [16]. The evidence indicates that homeodomain proteins are involved in antitumor immunotherapy in complex ways.

In this review, we focus on the role of HOPX (homeodomain-only protein X), a unique homeodomain protein, in tissue differentiation, metabolism, and immunity, along with carcinogenesis. HOPX is widely expressed in human tissues of the brain, heart, lung, placenta, intestine, skeletal muscle, bladder, spleen, kidney, and immune system. Unlike other homeodomain transcription factors, *HOPX* lacks DNA-binding capacity due to the absence of a DNA-binding domain. The distinctive structure of HOPX makes it act as a co-effector to regulate gene expression in multiple physiological and pathological processes.

## 2. Identification of the HOPX Gene

Initially, the homeodomain protein HOP, also named TOTO and Cameo (cardiac homeodomain), was identified in developing heart tissues by two research groups in September 2002, when the investigators performed a bioinformatics screen of expressed sequence tag (EST) databases using a homeodomain consensus sequence [17,18]. In the meantime, by screening the normal adult human gingival and cardiac cDNA libraries, an odd homeobox gene called odd homeobox 1 (*OB1*) which shared high similarity with HOP was identified in heart tissues (GenBank: AF492675.1, https://www.ncbi.nlm.nih.gov/nuccore/AF492675, accessed on 29 October 2025).

In 2003, Asanoma et al. [19] isolated a novel gene containing a homeodomain consensus motif from a PCR-based subtracted fragmentary cDNA library between normal placental villi and the choriocarcinoma cell line CC1. Since this gene was highly expressed in the placental tissues but not expressed in choriocarcinoma clone 1, it was named *NECC1*. Also in 2003, when we compared gene expression between normal human bronchial epithelial cells (HBEC) and a lung squamous cell carcinoma (SCC) cell line H2170 by using the suppression subtractive hybridization technique, our group cloned two cDNA libraries representing the genes that were upregulated and downregulated in HBEC and H2170, respectively [20]. A novel lung cancer-associated gene Y (*LAGY*), containing the homeodomain, was identified [21]. A complete loss of *LAGY* mRNA expression was observed in poorly differentiated lung cancer samples.

In 2004, Pauws et al. [22] performed serial analysis of gene expression (SAGE) to compare gene expression profiles of normal thyroid tissue and papillary thyroid carcinoma (PTC) and found that a SAGE tag corresponding to the partial cDNA for small protein 31 (*SMAP31*) was significantly upregulated in PTC. A homology search reveals that *SMAP31* possesses a homeodomain.

In 2006, using the Affymetrix platform, Spurlock et al. identified novel genes and physiological pathways that potentially facilitated beta-adrenergic receptor agonists (BA)-induced skeletal muscle growth [23]. Among the novel genes, there was an unusual homeobox only domain gene (*Hod*), which was notably upregulated in skeletal muscle tissues after BA administration.

In 2007, the *HOP* gene symbol was revised to *HOPX* (homeodomain-only protein X) to distinguish it from unrelated genes such as *hopscotch* in Drosophila and *hop-sterile* in mouse [24].

**Figure 1:**
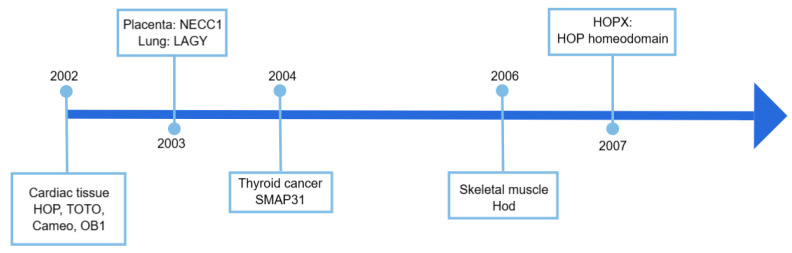
The timeline of the identification of the novel gene *HOPX* (*HOP*, *TOTO*, *OB1*, *NECC1*, *LAGY*, *SMAP31*, or *Hod*) in different types of tissues.

Since then, this homeodomain protein has been designated HOPX. The timeline of HOPX identification in different tissues is illustrated in Figure 1.

HOPX (also named HOP, TOTO, Cameo, NECC1, LAGY, SMAP31, or Hod, previously) is the smallest homeodomain protein, with 73 amino acids (AAs) containing a putative homeodomain motif. The human *HOPX* gene is localized on chromosome 4q11-q12, comprising seven exons and five mRNA transcripts generated by alternative splicing. HOPX has three different isoforms. Isoform A consists of 91 AAs, isoform B contains 73 AAs, and isoform C comprises 112 AAs [25]. Unlike other homeodomain transcription factors, HOPX lacks the critical residues essential for DNA-binding; thus, it is unable to bind to DNA. Instead, it acts as a co-effector interacting with other transcription factors to regulate gene transcription [17,18].

*HOPX* is highly conserved, and its homologs have been widely identified in vertebrates, including human, mouse, rat, pig, cow, chick, frog, and zebrafish [3]. In particular, the human HOPX protein shares 92% similarity with the mouse Hopx protein. In contrast to human *HOPX*, the mouse *Hopx* gene is located on chromosome 5, and its mRNA contains three splice variants encoding one Hopx protein with 73 amino acids.

## 3. HOPX in Tissue Differentiation and Proliferation

In mammalian bodies, there are four main tissue types, including epithelial, connective, muscle, and nervous tissue. Accumulating evidence supports the essential role of HOPX in tissue differentiation and organ development.

### 3.1. HOPX in Epithelial Tissue Differentiation and Development

HOPX is tightly involved in lung epithelium differentiation. During lung development, Hopx is expressed in mouse pulmonary alveolar type 1 (AT1) cells, and following lung injury repair, Hopx+ AT1 cells proliferate and trans-differentiate into AT2 cells [26]. The trans-differentiation of Hopx+ AT1 cells may give rise to 10% and 30% of the renewed AT2 cells in adult and neonatal lungs, respectively [27,28]. *HOPX* acts downstream of transcription factors *NK2 homeobox 1* (*Nkx2.1*) and *GATA6* to modulate lung epithelial differentiation and maturation. Loss of Hopx expression leads to defective AT2 development with increased surfactant production and disrupted lung architecture [29].

In the skin, Yang et al. found that HOPX was upregulated during human keratinocyte differentiation induced by phorbol 12-myristate 13-acetate (PMA), a protein kinase C (PKC) signaling activator, and *HOPX* siRNA knockdown significantly enhanced the expression of essential late-stage keratinocyte differentiation markers such as involucrin and loricrin in primary human keratinocytes [30]. Conversely, Obarzanek et al. observed that *HOPX* silencing decreased the calcium-induced terminal differentiation in HaCaT, an immortalized human skin cell line derived from keratinocytes, indicating a positive regulatory role of HOPX in epidermal differentiation [31]. This discrepancy might be caused by distinct genetic and epigenetic features of keratinocytes used as research models and different inducing systems used for differentiation.

Additionally, Hopx is expressed in the adult stem cell niche within the intestinal epithelium. It is a specific marker for cells in the +4 niche of the intestine, consisting of slow-cycling reserve stem cells, distinct from the stem cell niche situated at the crypt base containing crypt base columnar cells, which show a rapid dividing property [32]. HOPX is also expressed in differentiated colonic mucosal cells and involved in epigenetic regulation of differentiation-associated genes [33]. Additionally, using differentiated hepatocyte models induced by the hepatocyte-specifying regulator hepatocyte nuclear factor 4α (HNF4α) or blocked by stimulating the Notch signaling or interfering with the Smad signaling, HOPX was screened as one of the master regulators for hepatocellular differentiation [34].

### 3.2. HOPX in Connective Tissue Differentiation

Besides the expression in the keratinocytes of the epidermis, Hopx is found to be present in mouse hair follicles, which are composed of both epidermal (epithelial) tissue and dermal (connective) tissues. Hopx-positive cells are able to undergo self-renewal and provoke differentiated lineages of the hair follicles [31].

Abundant endothelial cells exist in the connective tissues. During placental development, HOPX induces the differentiation of trophoblast stem cells into the trophoblastic lineage in the chorionic connective tissue [35]. Hopx is expressed during hemato-endothelial lineage specification in vivo and is regulated by stem cell leukemia (SCL) during hemato-endothelial differentiation [36]. Loss of Hopx leads to damaged hematopoiesis partially through affecting Wnt/β-catenin signaling [36]. Increased HOPX expression is detected during bone marrow stromal cell (BMSC) differentiation. However, HOPX has no direct effect on the osteogenic capacity of BMSCs, since overexpression and siRNA knockdown of *HOPX* in BMSCs showed no effects on the mRNA expression levels of the master osteogenic regulator *RUNX family transcription factor 2* (*RUNX2*) and the mature bone marker *OSTEOPONTIN* (*OPN*). Instead, HOPX suppresses adipogenesis-associated genes, thus inhibiting BMSC adipogenic differentiation [37].

Moreover, HOPX takes part in the differentiation and development of lymphoid tissue, a specialized connective tissue. The role of HOPX in the differentiation and maturation of immune cells is depicted in Section 6, “HOPX and immunity”.

### 3.3. HOPX in Muscle Tissue Differentiation and Development

Initially, the role of Hopx in tissue differentiation and organ development was investigated in the developing heart of mice. HOPX, a downstream target of the transcription factor NK2 Homeobox 5 (Nkx2-5), antagonizes the activity of serum response factor (SRF) to modulate SRF-dependent cardiac-specific gene expression and heart muscle cell proliferation and differentiation [17,18].

Hopx recruits histone deacetylase (Hdac) activity to suppress SRF-dependent gene transcription [38]. Wild-type Hopx precipitates significant Hdac activity and forms a complex with HDAC2, while mutant HOPX does not. Both Hopx-transgenic and -knockout mice develop severe heart defects. Transgenic mice with Hopx overexpression develop hypertrophy, possibly through recruiting HDAC activity, which epigenetically suppresses anti-hypertrophic genes, while Hopx knockout mice exhibit a partially penetrant embryonic lethal phenotype due to a disruption of the balance between cardiac muscle cell proliferation and differentiation, leading to an abnormal growth of cardiomyocytes [18,38,39]. Additionally, in the developing mouse heart, Hopx integrates bone morphogenetic protein (Bmp) and Wnt signaling via interacting with activated Smads and repressing Wnt genes to promote cardiomyocyte differentiation [40]. However, as revealed by single-cell transcriptomic analysis, HOPX is rarely expressed during in vitro cardiac-directed differentiation from human pluripotent stem cells (PSCs) due to inactivation of hypertrophic signaling [41].

The regulatory role of HOPX is not limited to cardiac muscle cells. Using yeast two-hybrid screening for binding candidates, Kee et al. found that Hopx interacted with the transcription factor enhancer of polycomb1 (Epc1) to induce skeletal muscle differentiation [42].

### 3.4. HOPX in Nerve Tissue Differentiation

HOPX is one of the key players in neurogenesis, particularly in the differentiation of neural stem cells. During the development of the mouse brain, Hopx is spatially and temporally expressed in the central nervous system, including the cortex, cerebellum, dentate gyrus, etc. [43,44,45].

Li et al. observed that Hopx labeled quiescent neural stem cells (NSCs) in the adult hippocampus, and it was required for normal neurogenesis and NSC maintenance [44]. Hopx-null mice showed decreased activity of the Notch pathway. In the human fetus, HOPX is expressed in the indusium griseum (IG), a dorso-medial rudimentary part of the hippocampus, at midgestation, a critical period for its differentiation and reorganization [46]. This finding implies that HOPX plays a role in human neurodifferentiation and development. In line with this, HOPX has recently been identified as one of the regulators of differentiation and proliferation states in diffusely invading glioblastoma multiforme (GBM) cells. Knockout of HOPX in GBM led to a significant transition toward mesenchymal-like states, accompanied with decreased activation of developmental and proneural signatures [47].

## 4. HOPX in Metabolism

HOPX regulates metabolic programs associated with cardiomyocyte maturation. Friedman et al. observed that *HOPX* knockdown in cardiomyocytes resulted in a significantly reduced adenosine triphosphate (ATP) production, implying a HOPX-mediated shift to progenitor-like metabolic programs in the heart [48]. In the study, single-cell genomics and genome-wide footprinting, together with in vitro and in vivo genetic models, were applied. HOPX-dependent loci were found to be enriched in critical determinants of heart development and metabolism [48]. Additionally, under hypertrophic growth conditions, HOPX represses progenitor gene programs and upregulates structural and metabolic programs to promote the maturation of human-induced pluripotent stem cell (hiPSC)-derived cardiomyocytes [48]. In the murine alveolar epithelial cell line MLE12, knockdown of *Hopx* increased metabolic activity, as revealed by the WST-1 metabolic assay, based on the activity of mitochondrial dehydrogenases [49]. These data indicate a potential involvement of HOPX in metabolism.

The *HOPX* gene was identified as one of the downstream target genes of *Lipin-1*, a central regulator of lipid homeostasis [50]. In human Lipin-1-deficient myoblasts, HOPX was significantly upregulated. Recently, Hng et al. have depicted that under adipogenic inductive conditions, HOPX could not bind to enhancer of zeste homolog 2 (EZH2), an epigenetic factor which regulates HOPX during bone marrow stromal cell (BMSC) differentiation [37]. Based on “loss-of-function” and “gain-of-function” models, they demonstrated that HOPX inhibited adipogenesis in human BMSC via suppression of different adipogenic pathways and downregulation of adipogenic genes, including *ADIPOQ*, *FABP4*, *PLIN1*, and *PLIN4*, suggesting the impact of HOPX on overall lipid accumulation [37]. Moreover, it has been reported by Dmitrieva-Posocco et al. that the ketone body β-Hydroxybutyrate (BHB) suppresses colorectal cancer (CRC) progression by inducing HOPX [51]. BHB, an energetic metabolite produced during the ketogenic state, in which the primary energy source of the body shifts from glucose to fat-generating ketone bodies, plays a crucial role in glucose and lipid metabolism [52]. Exposing organoids from Hopx-deficient mice and wild-type littermates to BHB, the investigators found that BHB inhibited the growth of wild-type organoids, but not Hopx-deficient ones. Additionally, the inhibition of glucose metabolism failed to induce HOPX expression despite the effective suppression of cancer organoid growth. These data imply that BHB-mediated growth inhibitory effect in CRC cells requires HOPX, while glucose restriction-mediated CRC inhibition is not HOPX-dependent [51]. Moreover, using an organoid-based CRISPR screen, they identified *hydroxycarboxylic acid receptor 2* (*Hcar2*), a metabolic regulator, as one of the BHB target genes. Exposure of organoids from Hcar2-deficient mice to BHB failed to induce Hopx expression, indicating that BHB acts through Hcar2 to induce Hopx expression [51].

Recently, the human microbiome has been recognized as a central regulator of cancer biology, and a crosstalk between gut microbiotas and fatty acid metabolism can be frequently observed in cancer cells [53,54]. Considering that BHB mainly originates from body fats and HOPX acts as its downstream molecule, the role of HOPX in lipid metabolism can be speculated. However, the precise function of HOPX in lipid metabolism and microbial communities warrants further investigation.

## 5. HOPX and Carcinogenesis

### 5.1. Tumor-Inhibitory Function of HOPX

HOPX is essential for cancer biology. It is frequently downregulated in various types of cancer, including lung cancer [21,55,56,57], skin cutaneous melanoma [58], pancreatic cancer [59], gastric cancer [60], breast cancer [61,62], colorectal cancer (CRC) [63], glioblastoma [64], nasopharyngeal carcinoma (NPC) [65], hepatocellular carcinoma (HCC) [66], uterine endometrial cancer [67], head and neck cancer [68,69], thyroid cancer [70,71], choriocarcinoma [19], skin cutaneous melanoma (SKCM) [58], and esophageal cancer [72], suggesting a broad tumor-suppressive activity of HOPX in carcinoma. Additionally, many studies have revealed that the HOPX gene silencing is significantly related to DNA hypermethylation in cancer, indicating a common epigenetic regulatory mechanism of HOPX in tumor cells.

The tumor-suppressive function of HOPX can be exerted in different ways [73]. In human lung cancer, our research group found that HOPX exerted a tumor-suppressive function through Ras-induced senescence, and additionally, overexpression of HOPX led to a reduced activity of the AKT pathway and increased p53/p21 activity [56]. In lung adenocarcinoma (ADC) cells, HOPX and GATA6, two transcription factors, cooperatively limit the metastatic competence of ADC cells via modulating overlapping alveolar differentiation and invasogenic target genes [55]. In line with this, lung ADC patients with higher HOPX expression show a more favorable clinical outcome in comparison to patients with lower HOPX expression [56,74]. In spite of that, very recently, using single-cell chromatin accessibility profiling (scATAC-seq), Tian et al. [75] have identified two distinct drug-tolerant persister (DTP) subpopulations in epidermal growth factor receptor tyrosine kinase inhibitor (EGFR-TKI) models. *HOPX* was enriched in the DTP subpopulation related to the developmental pathway. Further experiments with *HOXP* knockdown cell models reveal that HOPX contributes to the osimertinib-induced DTP stage of lung ADC via regulating the NF-κB activation and repressing histone modifications [75]. This observation contradicts the role of HOPX as a tumor suppressor in lung ADC. The discrepancy might be related to different genetic features and epigenetic regulatory mechanisms of the lung ADC cell lines used for the experiments.

Similarly, the association of HOPX expression with drug efficacy was depicted in skin cutaneous melanoma (SKCM). HOPX expression is correlated to clinical sensitivity to the chemotherapy drugs cisplatin and paclitaxel, the kinase inhibitor nilotinib, and the PARP inhibitor veliparib, while it is related to drug resistance to the chemotherapy drug gemcitabine and the mitogen-activated protein kinase kinase (MEK) inhibitor trametinib [58]. Again, this may reflect the complex and context-dependent functions of HOPX in cancer development and therapy response. Additionally, HOPX promotes apoptosis and S-phase arrest in SKCM cells.

Indeed, induction of cell cycle arrest and apoptosis is one of the important mechanisms through which HOPX exerts its tumor suppressive function. In pancreatic carcinoma, HOPX positively regulates the subG1 and G0/G1 phases and therefore reduces DNA synthesis and promotes cell death [59]. Similarly, in gastric cancer and breast cancer, HOPX inhibits cell proliferation, colony formation, and invasion and promotes apoptosis [60,61,62]. Furthermore, in patients with breast cancer, *HOPX* DNA methylation is significantly related to human epidermal growth factor receptor 2 (HER2) positivity and advanced lymph node metastasis, and it predicts an unfavorable clinical outcome [62]. *HOPX* DNA methylation is associated with worse prognosis of stage III CRC patients. Overexpression of HOPX results in enhanced apoptosis, reduced proliferation, invasion, and anchorage-dependent growth in vitro and inhibits tumorigenesis and angiogenesis in a mouse xenograft model [63]. In head and neck squamous cell carcinoma (HNSCC), overexpression of HOPX leads to decreased cell proliferation and enhanced sensitivity to UVA-induced apoptosis [62]. In human GBM, De Toni et al. found that downregulation of HOPX decreased the number of apoptotic cells and increased the number of newly formed granule neurons, while overexpression of HOPX resulted in increased apoptosis and decreased tumorigenic ability in a subset of GBM spheroids [64]. In nasopharyngeal carcinoma (NPC) and hepatocellular carcinoma (HCC) cells, HOPX overexpression inhibits tumor cell migration, invasion, and metastasis via targeting the SNAIL transcription factor [65,66]. Recently, it has been reported that the methyltransferase-like 3-mediated m6A modification of miR-1908-5p contributes to NPC progression by targeting HOPX [76]. In human uterine endometrial cancer, overexpression of HOPX decreases cell proliferation, tumorigenicity, and c-fos expression in response to 17-β-estradiol stimulation via inhibiting SRF [67]. Hypermethylation of the *HOPX* DNA was identified in patients with thyroid cancer, associated with recurrent/progressive disease and poor clinical outcome [70,71]. In choriocarcinoma, ectopic HOPX expression results in cell morphological changes and inhibition of in vivo tumorigenesis. HOPX may be involved in directing villous morphogenesis and regulating invasiveness by induction of trophoblast fusion and terminal differentiation [19]. Using patient-derived esophageal squamous cell carcinoma (ESCC) samples and mouse models, Georgy et al. depicted that upregulation of HOPX by transcription factor grainyhead-like protein 3 homolog (GRHL3) suppressed tumor cell growth via inactivation of the Wnt/β-catenin signaling [72].

### 5.2. Tumor-Promoting Function of HOPX

As described above, a body of evidence supports the notion that HOPX serves as a tumor suppressor in various types of carcinomas. However, several studies have also pointed out the pro-tumorigenic effect of HOPX. RNA-seq analysis shows that *HOPX* is upregulated in intrahepatic cholangiocarcinoma tissues compared to adjacent noncancerous hepatic tissues [77]. Pavlova et al. demonstrated that *HOPX* knockdown in cutaneous squamous cell carcinoma (SCC) cells accelerated apoptosis, evident by an elevated activity of caspases-3/7, downregulation of pro-survival proteins like BCL2 (B-cell leukemia/lymphoma 2 protein) and MCL1 (induced myeloid leukemia cell differentiation protein), and an upregulation of pro-apoptotic effectors including BAX (BCL2 associated X) and BAK (BCL2 antagonist/killer 1), BH3-only proteins, and caspase-8 [78]. This indicates that loss of HOPX activates both intrinsic and extrinsic apoptotic pathways. In 347 adult patients diagnosed with de novo acute myeloid leukemia (AML), Lin et al. observed that HOPX expression was associated with distinct clinical and molecular features such as higher platelet counts, lower white blood cell counts, lower lactate dehydrogenase levels, and mutations in the *RUNX1* (*RUNX family transcription factor 1*), *IDH2* (*isocitrate dehydrogenase 2*), *ASXL1* (*ASXL transcriptional regulator 1*), and *DNMT3A* (*DNA methyltransferase 3 alpha*) genes. In addition, higher expression of HOPX predicted lower complete remission, as well as shorter survival [79]. Consistent with this finding, He et al. [80] described the oncogenic function of HOPX in AML cells. Interacting with HDAC2, HOPX induced AML differentiation blockage and malignant progression. Interestingly, the same group (Lin et al.) later found that in T cell acute lymphoblastic leukemia (T-ALL), reduced HOPX expression was related to increased tumor burden, suggesting its tumor-suppressive role in T-ALL [81]. The divergent tumor biological behaviors of HOPX in AML and T-ALL imply the context-dependent function of HOPX in hematologic oncology. It is tempting to speculate that genetic characteristics, such as increased tumor mutational burden, might contribute to the tumor-promoting function of HOPX. Future studies including genomics analysis will provide crucial insight into the molecular mechanism of HOPX in specific malignancy.

**Figure 2:**
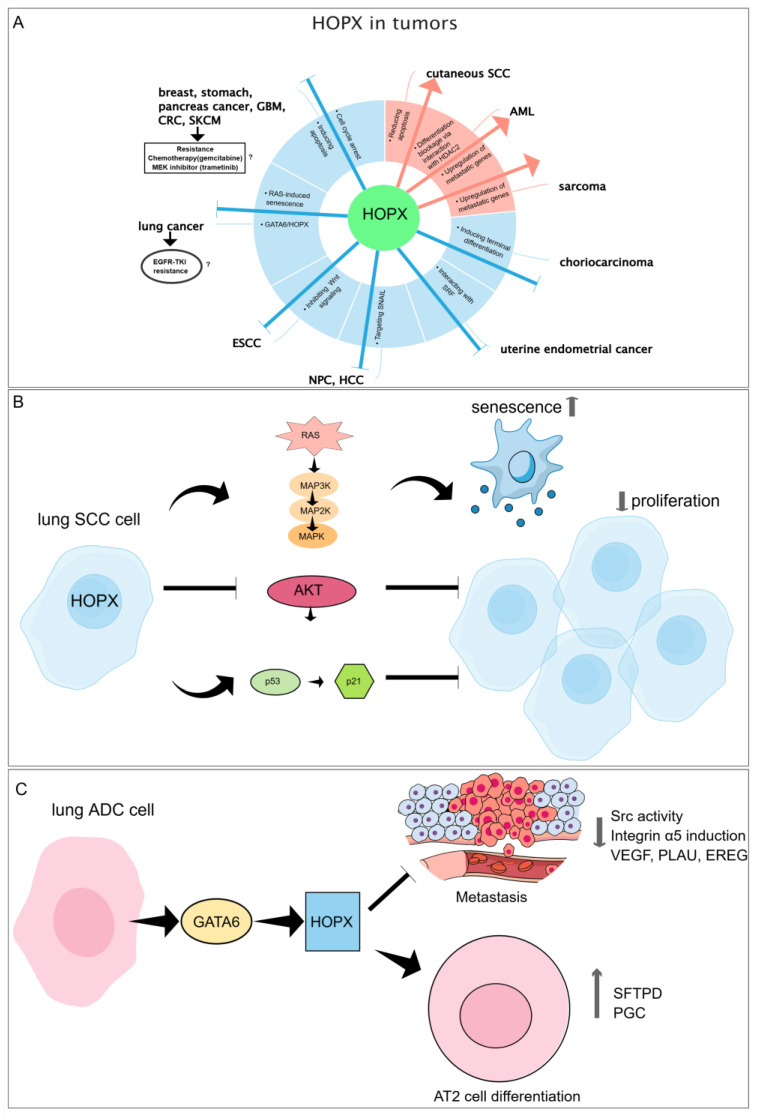
The roles of HOPX in various types of tumors and its mechanisms of action in lung cancer cells. (**A**) The tumor-suppressive function (highlighted in blue) and the oncogenic function (highlighted in red) of HOPX in tumors. The association between HOPX and clinical sensitivity to anticancer drugs in SKCM and lung cancer is indicated. The mechanisms of action of HOPX in (**B**) lung squamous cell carcinoma (SCC) cells and (**C**) lung adenocarcinoma cells. HOPX inhibited SCC cell proliferation by increasing Ras-induced cellular senescence, inactivating the AKT pathway, and enhancing the p53/p21 activity. HOPX interacting with GATA6 inhibits the ADC cell metastasis and promotes alveolar type II (AT2) cell differentiation, accompanied by downregulation of metastasis-associated genes and upregulation of lung differentiation-associated genes. GBM: glioblastoma multiforme; CRC: colorectal cancer; SKCM: skin cutaneous melanoma; ESCC: esophageal squamous cell carcinoma; NPC: nasopharyngeal carcinoma; HCC: hepatocellular carcinoma; AML: acute myeloid leukemia; MEK: mitogen-activated protein kinase kinase; EGFR-TKI: epidermal growth factor receptor (EGFR)–tyrosine kinase inhibitor; ADC: adenocarcinoma.

In mesenchymal cell-derived sarcoma, HOPX functions as an oncogene. Using the v-src-transformed metastatic cell line PR9692, Kovarova et al. showed that *HOPX* knockdown decreased the in vivo metastatic capacity in a syngeneic animal model system, along with significant alterations of metastatic-associated genes such as *NCAM* (*neural cell adhesion molecule*), *FOXG1* (*forkhead box G1*), and *ITGA4* (*integrin subunit alpha 4*) [82]. The role of HOPX in human cancer is shown in Figure 2.

### 5.3. HOPX in the Tumor Microenvironment (TME)

Using single-cell RNA sequencing (scRNA-seq) of fibroblast cells derived from 517 human samples under diverse pathological conditions, Gao et al. identified distinct fibroblast subpopulations [83]. *HOPX*-overexpressing myofibroblasts are enriched in nephrosclerosis, a kidney disease characterized by interstitial fibrosis, and several cancerous tissues, including pancreatic and ovarian cancer [83]. Fibroblasts with HOPX signature upregulate fibrosis-associated genes, as well as genes involved in adipogenesis and mesenchymal stem cell differentiation, indicating a potential role of HOPX in modulating the TME. Indeed, *HOPX* is considered one of the cancer-associated fibroblast (CAF)-related genes, and high expression of HOPX was significantly associated with shorter metastasis-free survival of patients with prostate carcinoma [84]. However, in colorectal carcinoma, HOPX was found to be highly expressed in normal stromal cells from the mucosa in comparison with desmoplastic tumor stromal cells within the TME [63]. In the analysis of the immune and metabolic profiles of GBM organoids from patient-derived glioblastoma stem cells (GBMS), an immune-like molecular program including cytokines, antigen presentation and processing, T cell receptor inhibitors, and interferon regulatory genes was found to be significantly related to HOPX+ and special AT-rich sequence-binding protein 2 (SATB2+) progenitor populations [85]. Additionally, using scRNA-seq and bulk RNA-seq, Zhan et al. identified a novel monocyte/macrophage-related gene signature for the prediction of the clinical outcome and immune response in AML [86]. They found that the signatures of eight monocytes/macrophages, including *HOPX*, were related to shorter survival time and predicted drug sensitivity to the PARP inhibitor ABT-888 and the tyrosine kinase inhibitor Axitinib in patients with AML.

The divergent expression of HOPX in the TME across different tumor types might be due to several factors, including alterations of tumor cells and stromal cells driven by genetic and epigenetic mechanisms, as well as complex interactions of tumor cells with immune cells and fibroblasts. These might in turn affect tumor therapy efficacy.

## 6. HOPX and Immunity

### 6.1. Expression of Hopx in Cells of the Immune System

The detection of Hopx in immune cells can be traced back to the publications by Albrecht et al. and Hawiger et al. in 2010 [87,88]. Albrecht et al. observed that Hopx expression increased with the progression of naive T helper (Th) cells to an effector/memory phenotype in murine models. In line with this, *HOPX* expression was found to be low in naïve Th cells, while it was increased in terminally differentiated effector/memory Th1 cells isolated from healthy human donors [87]. This suggests that *HOPX* may be a marker for effector/memory Th1 cells. Additionally, it is found that Hopx plays a critical role in effector/memory Th1 cell survival through the regulation of genes involved in Fas-mediated apoptosis [87]. Hawiger et al. found that Hopx was required for the function of regulatory T (Treg) cells in dendritic cell-induced peripheral T cell unresponsiveness [88]. The possible mechanism is that hopx decreases the expression of the activator protein 1 (AP-1) complex that positively regulates interleukin-2 (IL-2). Inhibition of IL-2 contributes to Treg cell survival [89].

**Figure 3:**
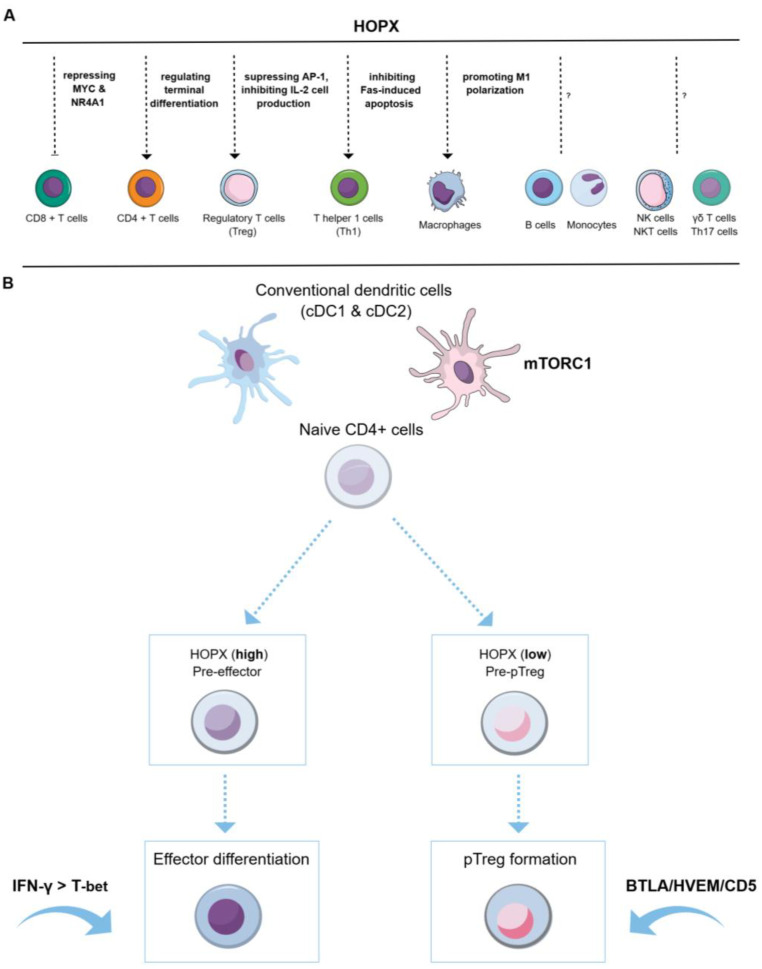
The expression and function of HOPX in the cells of the immune system. (**A**) HOPX expression and its reguratory mechanisms in immune cells. HOPX is present in the majority of the immune cells and serves as a negative regulator of CD8+ T cell activation through repressing the transcription factors MYC and NR4A1 to suppress excessive T cell responses. HOPX regulates the terminal differentiation of CD4+ T cells. HOPX promotes the functional Treg cells by suppression of AP-1, which in turn decreases the expression of IL-2, leading to the survival of functional Treg cells. HOPX enhances Th1 cell survival by inhibition of Fas-induced apoptosis. HOPX enhances macrophage functions by promoting M1 polarization. (**B**) CD4+ T cell fate determination is related to the expression of HOPX. The presentation of cDC1 and cDC2 together with mTORC1 induces HOPX (high) pre-effectors. Terminal efffetor differentiation is mediated by IFN-γ which promotes the expression of the transcription factor T-bet. The presentation of cDC1 mainly induces HOPX (low) pro-pTreg cells. The conversion from pro-pTreg cells to terminal pTreg cells is mediated by the BTLA/HVEM/CD5 axis.

Besides the expression of *Hopx* in Th1 and Treg cells, *Hopx* was detected in other mature immune cells such as effector CD4+ and CD8+ T cells, Th17 cells, B cells, natural killer (NK) cells, NKT cells, effector/memory T cells, and macrophages (Figure 3A), but it was absent in naïve CD4+ cells, dendritic cells, and eosinophils, based on flow cytometry and transcriptome analyses [90,91,92,93,94,95,96].

### 6.2. Involvement of Hopx in Immune Cell Proliferation and Differentiation

Expression of *Hopx* was detected in hematopoietic stem cells (HSCs), the foundational cells of the blood and immune system. Transcriptomic study of the Hopx−/− bone marrow cells shows significant downregulation of the C-X-C motif chemokine ligand 12 /C-X-C motif chemokine receptor 4 (Cxcl12/Cxcr4) axis, which is critical for maintenance of HSC quiescence, indicating an important role of HOPX in sustaining the long-term self-renewal and multi-lineage differentiation potential of HSCs [97].

In immune cells, the early induction of *Hopx* is associated with the transition from naïve T cells to specialized CD4+ T cells, and the early expression of *Hopx* implies the onset of immune effector processes, including antigen-specific activation and cytokine-driven polarization [98]. Based on the distinct expression levels of Hopx, activated T cells can be split into two types: Hopx-overexpressing “pre-effectors” and Hopx-underexpressing potential Treg cells. Hopx-overexpressing “pre-effectors” emerged under a homeostatic state can differentiate into autoimmune effector T cells, which participate in autoimmune responses and tumor immunosurveillance [99,100], while Hopx-underexpressing potential Treg cells will convert into terminal pTreg cells [98,100]. Opejin et al. [100] found that the induction of Hopx-overexpressing effector precursors could be promoted by the mammalian target of rapamycin complex 1 (mTORC1) and type 2 dendritic cells (cDC2). In this process, programmed production of interferon-gamma (IFN-γ) and expression of the transcription factor T-bet promoted by IFN-γ are involved. The terminal pTreg conversion can be mediated by cDC1 and cDC2, and for the cDC1-specific pTreg conversion, the function of B and T lymphocyte associated/herpesvirus entry mediator/CD5 (BTLA/HVEM/CD5) axis is important [100]. BTLA recruits HVEM expressed in CD4+ T cells and increases CD5 expression in CD4+ T cells to stimulate pTreg cell conversion [98,100]. The role of HOPX in CD4+ T cell fate determination is illustrated in Figure 3B.

### 6.3. Hopx in Differentiated CD4+ and CD8+ Cells, as Well as NK Cells: A Potential Enhancer in Anticancer Immunity

Hopx is expressed in murine CD8+ and CD4+ cells under steady-state conditions. Using single-cell RNA sequencing, Bourque et al. found that approximately 60% of CD8+ T cells in the murine spleen and lymph nodes expressed *Hopx*, while no more than 20% of CD4 + Foxp3^neg^ T cells expressed *Hopx* [90]. In human CD4+ T and CD8+ T cells, the expression of *HOPX* was also detected, with the isoform B presenting the dominant one [101,102].

Yang et al. observed that transduction of HOPX in both CD8+ and CD4+ T-naïve cells with in vitro stimulation by anti-CD3 and anti-CD28 antibodies led to significantly decreased cell growth, suggesting the role of HOPX in T cell proliferation and differentiation [101]. Moreover, by microarray analysis and chromatin immunoprecipitation coupled with quantitative PCR, they found that HOPX exerted an anti-proliferative effect in CD8+ T cells via suppressing the expression of the T cell proliferative regulators MYC and nuclear receptor subfamily 4 group A member 1 (NR4A1). This negative regulatory mechanism of HOPX may prevent excessive T cell responses that could lead to self-attacks in human diseases.

Under cancerous conditions, increased HOPX was detected in CD4+ and CD8+ T cells, and HOPX overexpression in cancer cells is significantly related to CD8+ T cell infiltration in the TME. Transcriptomic analysis revealed that the majority of neoantigen-reactive CD4 + T cells from a patient with bile duct cancer expressed HOPX [103]. Additionally, in patients with renal cell carcinoma showing partial response to immune checkpoint therapy, higher expression of HOPX was detected in CD4+ tumor-infiltrating lymphocytes (TILs) compared to patients with progressive disease [103]. By GO/KEGG analysis, GSVA, immune scoring, and single-cell sequencing analysis, He et al. found that HOPX was highly enriched in CD8+ T cells within the TME of skin cutaneous melanoma, and high expression of HOPX in tumor cells was significantly associated with CD8+ T cell infiltration [58]. Moreover, in gastric cancer, using mouse models, Yu et al. showed that CD8+ T cell cytotoxicity was enhanced by gut microbial butyrate via the G protein-coupled receptor 109A (Gpr109a)/Hopx axis [104]. Upon stimulation with cytokines, natural killer (NK) cells differentiate into cytokine-induced memory-like (ML) NK cells that acquire enhanced cytolytic activity. Using single-cell transcriptomes of NK cells isolated from leukemia patients after adoptive transfer, *HOPX*, together with other NK cell receptor repertoires, is found to be highly expressed in ML NK cells, which exhibit more persistent antitumor efficacy [105,106]. This hints the potential of HOPX in defining the memory NK cell state and promoting survival of ML NK cells.

As mentioned above, in physiological conditions, HOPX acts as a molecular brake, suppressing proliferation of CD8+ cells by repressing key cell cycle regulators like MYC and NR4A1 [101], while under tumorous conditions, expression of HOPX in CD8+ T cells is associated with CD8+ T cell infiltration and enhanced antitumor immunity. The mechanisms of action of HOPX contributing to distinct functions in CD8+ T cells deserve further investigation. Nevertheless, in the context of tumors, HOPX might facilitate the rapid enrichment of CD8+ T cells, neoantigen-reactive CD4+ T cells, and possibly their corresponding receptors within the TME, which in turn boosts the immune system to combat cancer.

## 7. Conclusions and Future Perspectives

The transcription factor HOPX is the smallest homeodomain protein with a unique feature. It lacks a DNA-binding domain and thus functions as a co-effector to regulate gene transcription. The interactions of HOPX with other transcription factors are specific, suggesting that its role might not be redundant or easily replaced by other homeodomain proteins.

Since the full-length cDNA and the protein structure of HOPX were identified in 2002 in cardiac tissues, growing evidence has demonstrated the multifaceted functions of HOPX in tissue differentiation, proliferation, and carcinogenesis across various tissues and developmental processes. Recently, its emerging roles in metabolism and autoimmune responses have gained attention. HOPX is expressed in cancer-associated fibroblasts (CAFs) and immune cells, two major components of the tumor microenvironment (TME), indicating its potential in modulating the TME.

Over the past two decades, the biological functions of HOPX in malignant tumors have been extensively investigated. However, for the development of personalized therapeutic strategies by targeting HOPX, there is still a long journey ahead. Further studies focusing on the identification of the HOPX-mediated signaling pathways, characterization of its tumor cell-specific functions, and evaluation of its influence on drug sensitivity are required. Clarifying its mechanisms of action will help to stratify patients for individualized therapy.

On the other hand, the precise functions and regulatory mechanisms of HOPX in tumor metabolism and immunity need to be explored. Current multi-omics technologies offer a comprehensive understanding of gene function by integrating data from different biological layers, and particularly at the single-cell level. Defining the function of HOPX in metabolism and crosstalk between tumor cells and the TME components will be essential for the development of strategies for targeted therapy and anticancer immunotherapy.

## Abbreviations

The following abbreviations are used in this manuscript:

AAs	Amino acids
ADC	Adenocarcinoma
AML	Acute myeloid leukemia
BTLA/HVEM	B and T Lymphocyte Associated/herpesvirus entry mediator
CAF	Cancer-associated fibroblast
CD8	Cluster of differentiation 8
CRC	Colorectal cancer
EGFR-TKI	Epidermal growth factor receptor tyrosine kinase inhibitor
FOXG1	Forkhead box G1
GATA6	GATA binding protein 6
GBM	Glioblastoma multiforme
GRHL3	Grainyhead-like 3
HOPX	Homeodomain only protein X
HSC	Hematopoietic stem cell
ITGA4	Integrin subunit alpha 4
NCAM	Neural cell adhesion molecule
NK cells	Nature killer cells
NPC	Nasopharyngeal carcinoma
NSC	Quiescent neural stem cells
SCC	Squamous cell carcinomas
scRNA-seq	Single-cell RNA-sequencing
SKCM	Skin cutaneous melanoma
SRF	Serum response factor
T-ALL	T cell acute lymphoblastic leukemia
Th cells	T helper cells
TME	Tumor microenvironment
Treg cells	Regulatory T cell
